# An X‐Ray Absorption Spectroscopy Investigation into the Fundamental Structure of Liquid Metal Alloys

**DOI:** 10.1002/smsc.202400317

**Published:** 2024-09-23

**Authors:** Jaydon A. Meilak, Karma Zuraiqi, Valerie Mitchell, Bernt Johannessen, Brittany V. Kerr, Pierre H. A. Vaillant, Krystina Lamb, Patjaree Aukarasereenont, Caiden Parker, Taren Cataldo, Francois Malherbe, Andrew J. Christofferson, Torben Daeneke, Rosalie K. Hocking

**Affiliations:** ^1^ School of Science, Technology and Engineering Swinburne University of Technology John St Hawthorn VIC 3122 Australia; ^2^ Royal Melbourne Institute of Technology 124 Latrobe St Melbourne VIC 3000 Australia; ^3^ Australian Synchrotron 800 Blackburn Rd Clayton VIC 3168 Australia

**Keywords:** gallium, liquid metals, X‐ray absorption spectroscopy

## Abstract

Gallium and gallium alloys have gained significant interest due to gallium's low melting point. This property allows for gallium‐based catalysts to take advantage of the unique reaction environments only available in the liquid state. While understanding of the catalytic properties of liquid metals is emerging, a comprehensive investigation into the fundamental structures of these materials has yet to be undertaken. Herein, the structure of liquid gallium, along with related liquid alloys EGaIn, EGaSn, and Galinstan are explored using X‐ray absorption spectroscopy (XAS). In contrast to some other studies that show dimers, analysis of the XAS data both in X‐ray absorption near edge structure and extended X‐ray absorption fine structure shows that when fully dissolved the materials are largely homogenous with no obvious signs of local structures. Ga shows a bond contraction when melted which is consistent with its increase in density; however, an expansion in bond length is observed when alloyed with In and Sn. XAS data indicate that the effective nuclear charge (*Z*_eff_) of In and Sn follows the trend expected based on electronegativity. Molecular dynamic (MD) simulations are performed to simulate the structure and trends between MD and XAS; the trends agree well but MD overestimates bond lengths.

## Graphical abstract

Ga and Ga alloys are of significant interest as liquid metal catalysts. However, less is known about the structural environment that exists in the liquid state. Herein, X‐ray absorption spectroscopy is utilized at each Ga K, In K, and Sn K‐edge to learn about the structure of the liquid metals.

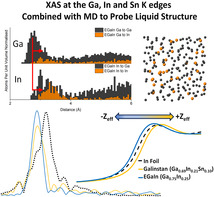

## 1 Introduction

Gallium (Ga) is a metal that melts at 29.7 °C;^[^ ^1^ ^]^ this along with its ability to form low‐temperature liquid metal alloys^[^ ^2^ ^]^ and the fact it is relatively nontoxic^[^ ^3^ ^]^ make it an interesting candidate for innovation in catalysis. Alloys such EGaIn (Ga 75 wt%, In 25 wt%), EGaSn (Ga 91 wt%, Sn 9 wt%), and Galinstan (Ga 68.5 wt%, In 21.5 wt%, and Sn 10 wt%) have been found to be important in key reactions including methanol oxidation,^[2e]^ graphene synthesis,^[^ ^4^ ^]^ coke‐resistant alkane dehydrogenation,^[^ ^5^ ^]^ and ammonia synthesis.^[^ ^6^ ^]^

Research into gallium's reactivity has grown significantly in the last two decades, but details about the atomic organization in gallium and its alloys remain largely unexplored. This is, in part, because liquid metals are very difficult to study by traditional analytical techniques due to their high density and lack of transparency which makes many types of spectroscopic characterization challenging.^[^ ^7^ ^]^ X‐ray absorption spectroscopy (XAS) is an element‐specific technique capable of probing the average electronic structure of a target element and determining its average local geometric structure from that element outward, which is done through analysis of the X‐ray absorption near edge structure (XANES) and extended X‐ray absorption fine structure (EXAFS), respectively, thus allowing for XAS to be used on noncrystalline,^[^ ^8^ ^]^ multimetal,^[^ ^9^ ^]^ and liquid systems.^[^ ^10^ ^]^ XAS has been applied to many functional and complex systems to provide geometric and electronic information^[^ ^11^ ^]^ for both in situ and ex situ studies of catalysts,^[^ ^12^ ^]^ soils,^[^ ^13^ ^]^ and even liquids such as water^[^ ^14^ ^]^ and Hg.^[^ ^15^ ^]^

**Figure 1:**
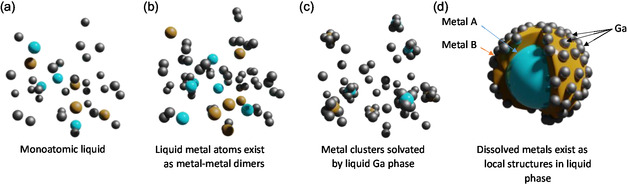
Possible arrangements in a liquid metal solution: a) a homogenous liquid metal, b) metal dimers, c) there is preferential ordering or bias in how the liquid metal exists, and d) metal colloids, nanoparticles, or clusters are dissolved in gallium.

When considering the local structure and the type of bonding that may occur within a liquid metal system, several structures have been proposed,^[^ ^1^, ^16^ ^]^ as illustrated in **Figure** **1**. They include a homogenous distribution of free‐floating liquid atoms (Figure 1a); liquid metal dimers (Figure 1b); solvated metals (Figure 1c); or as small metal clusters or colloids (Figure 1d). Understanding the distinction between these structures is fundamental for understanding mechanisms by which these materials function.

Herein, we use XAS to probe the nature of liquid metals focusing on gallium and its common liquid metal alloys, EGaIn, Galinstan, and a EGaSn, to understand how elements in these systems mix and how this mixing contributes to stabilizing the alloy. Previous studies by other research groups using X‐ray diffraction,[^16^, ^17^] neutron diffraction,^[^ ^17^, ^18^ ^]^ and atomic pair distribution function^[^ ^19^ ^]^ with Reverse Monte Carlo^[^ ^20^ ^]^ have been conducted on liquid Ga to help understand various components of the liquid state with some work suggesting that at elevated temperatures (1000 °C) Ga may exist in diatomic structures^[^ ^21^ ^]^ and others suggesting that while in the liquid state Ga resembles GaII (bcc) or GaIII (fcc) crystal phases.^[^ ^22^ ^]^ As such, analysis to identify the possibility of an internal structure to these liquids such as Ga–Ga dimers or crystal structures was a key part of our present study.

Molecular dynamic (MD) simulations were explored alongside the XAS and used to help model the XAS data.

## 2 Results and Analysis

### 2.1 XANES Analysis of Liquid Metal Alloys

**Figure 2:**
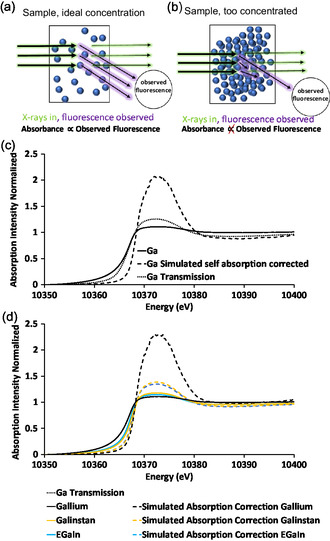
a) An illustration of a typical XAS experiment using an ideal sample showcasing data being collected in both fluorescence and transmission modes. b) An illustration of an experiment where the sample is not ideal, either too thick or concentrated resulting in the light absorbed and fluorescence not being proportional to one another thus altering the results. c,d) XANES of the Ga K‐edge of the major alloys. (c) Data on liquid gallium with a sample taken in transmission and another in fluorescence detection modes alongside the theoretic self‐absorption corrected data. (d) The gallium K‐edge of each of each gallium, Galinstan, and EGaIn; a comparison of each alloy and self‐absorption correct correction versus the thick Ga, Ga self‐absorption, and transmission scan.

An X‐ray absorption experiment fundamentally measures absorbance as a function of energy either directly through transmission or indirectly through the proportionality between fluorescent X‐rays and transmitted X‐rays (**Figure** **2**a). The resulting XAS spectra consists of two main regions: the XANES region and the EXAFS region which provide complementary information. XANES largely consists of bound state transitions and thus is reflective of electronic structure,^[^ ^8^ ^]^ the XANES energy position is often highly correlated with effective nuclear charge or redox state.^[^ ^8^ ^]^ The EXAFS intensity is associated with the energy dependence of electron interference and is largely reflective of the geometry around an absorbing atom. One of the challenges with collecting XAS data on liquid metals is sample concentration.

As discussed, XAS data may be collected in either transmission or fluorescence detection modes (Figure 2a). Transmission mode necessitates the sample being X‐ray transparent while fluorescence mode does not. Most liquid metals require substantial dilution to achieve X‐ray transparency; however, we found dilution by making thin films resulted in particle agglomeration. Therefore, fluorescence mode was used to avoid the need for dilution and the resulting agglomeration effects. They can be studied using fluorescence; however, the concentration of the metals is a factor that will impact the proportionality between the absorbance and the fluorescence resulting in self‐absorption.^[^ ^8^, ^23^ ^]^ Self‐absorption, in general, is a well‐documented and understood phenomena,^[^ ^24^ ^]^ and as such several steps can be taken to eliminate or account for it in a dataset. One common method used to remove the effects of self‐absorption experimentally is to dilute the sample with a dilutant that has a low Z number and is relatively inert such as cellulose^[^ ^25^ ^]^ or boron nitride (BN).^[^ ^26^ ^]^ In the work, we attempted many ways to eliminate self‐absorption of liquid metals. What we found was that liquid metals do not easily stay dispersed with common XAS dilutants such as BN and cellulose.

Self‐absorption (SA) can, to some extent, also be corrected for by calculation using the methods in references Goulon et al. and Tröger et al.^[^ ^27^ ^]^ and is further discussed in the SI with simulations shown below and in Figure S2, Supporting Information. Figure 2c shows simulated SA corrections of a 100% Ga scan (dashed), uncorrected Ga scan taken in fluorescence mode (dashed), and a transmission scan of a thin piece of Ga (dotted). As can be seen in Figure 2c, artificial self‐absorption correction of pure Ga results in an a substantially stronger XANES than observed in transmission scan (black dotted). We believe this was partially due to the Ga reaggregating and not staying properly dispersed. The three alloys taken in their natural form by fluorescence are shown in Figure 2d along with their self‐absorption correction. The uncertainties in the XANES positions associated with Ga are clear and described further below.

**Figure 3:**
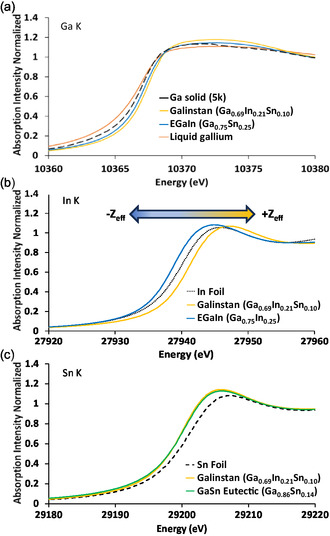
XANES at the a) Ga K‐edge, gallium liquid (orange) and solid (black dashed) EGaIn (blue) and Galinstan (yellow), b) In K‐edge of EGaIn (blue), Galinstan (yellow), and In foil (black dashed), and c) Sn K‐edge of EGaSn (green), Galinstan (yellow), and Sn foil (black dashed).

**Figure** **3** shows the XANES spectra at the Ga, In, and Sn K‐edges of four liquid metal samples—Ga, EGaIn, EGaSn, and Galinstan in a liquid state along with their solid metallic counterparts.

The Ga K‐edge of liquid and solid gallium, liquid EGaIn, and liquid Galinstan are plotted in Figure 3a. As shown, a clear shift can be seen in the rising edge position to lower energy in liquid Ga compared to the solid form, as well as in liquid EGaIn and Galinstan (Figure 3a). On the surface these shifts may suggest a higher effective nuclear charge (*Z*_eff_) of Ga present in these systems. However, it should be noted that the Ga K‐edge is substantially affected by self‐absorption (Figure 3a) and as such these trends may not be indicative of the effective nuclear charge of Ga in this system.

Figure 3b compares the In K‐edge of EGaIn and Galinstan; when compared to the In foil, a shift toward higher energy in Galinstan and a shift toward lower energy in EGaIn are observed. This suggests an increase in the effective nuclear charge of In in Galinstan and a decrease in EGaIn relative to the reference In foil. Examinations of the Ga and In K‐edges of EGaIn are consistent and show that a charge transfer from Ga to In occurs in this system. However, when observing the In and Ga K‐edges in Galinstan, a shift to higher energy relative to the metal reference is seen at both edges, indicating that both In and Ga may have higher *Z*_eff_. In comparison, Sn appears to have a lower *Z*_eff_, indicating the electrons spend more time around Sn relative to In and Ga. This could be due to a net electron movement or the consequences of bonding, or both.

To understand what is occurring to the Sn in Galinstan, the Sn K‐edge was measured and is shown in Figure 3c. The Sn K‐edge shows a shift to lower energy when compared to a Sn foil in both Galinstan and a binary EGaSn liquid system. This visible shift toward lower energy is indicative of Sn gaining electrons from both Ga and In. As shown by the XANES spectra at the Ga, In, and Sn K‐edges in binary systems (such as EGaSn and EGaIn), the Ga *Z*_eff_ appears to increase while that of In and Sn decreases. However, in systems containing all three metals Ga and In appear to gain effective nuclear charge, while only the charge of Sn decreases. This suggests that Ga is the most donating followed by In, with Sn being the most accepting based on Sn and In XANES shifts alone.

### 2.2 MD Simulations

To understand both the XANES and EXAFS results, MD simulations were performed. There are two ways the outcomes from these calculations could be examined for comparison with the XAS results. *Z*_eff_ on the atoms can be calculated using Bader charge analysis and the trends compared to the XANES shifts noted above. Additionally, the distribution of distances can be calculated and compared to those determined from EXAFS.

Due to the way the self‐absorption effects impact Ga XANES energy it is only valid to compare the shifts seen in Sn and In, as the error bar on the Ga‐edges is impacted by self‐absorption making it difficult to interpret edge energies. Table S4, Supporting Information displays the Bader charge analysis calculated from the MD simulations. From these charges, we see the trend most reduced/electron accepting Ga<Sn<In most oxidized electron donating. Analysis of the In and Sn XANES can allow us to probe the trends seen in the MD calculations. When comparing the In K‐edge in Galinstan to the In K‐edge in EGaIn, In appears more oxidised in Galinstan as denoted by the shift towards higher energy. Analysis of the Sn K edge of Galinstan shows the opposite trend with a shift towards lower energy indicating a more reduced state. This relative oxidation of In and reduction of Sn in Galinstan is consistent with a charge transfer from In to Sn as seen in the Bader charges shown in Table S4, Supporting Information. These trends match the electronegativity of In (1.78)^[^ ^28^ ^]^ and Ga (1.81)^[^ ^28^ ^]^ being very similar and lower than Sn (1.96)^[^ ^28^ ^]^ and the electron affinity of Ga (28.9 kJ mol^−1^),^[^ ^29^ ^]^ In (29.2 kJ mol^−1^),^[^ ^29^ ^]^ and Sn (107.3 kJ mol^−1^).^[^ ^29^ ^]^

**Figure 4:**
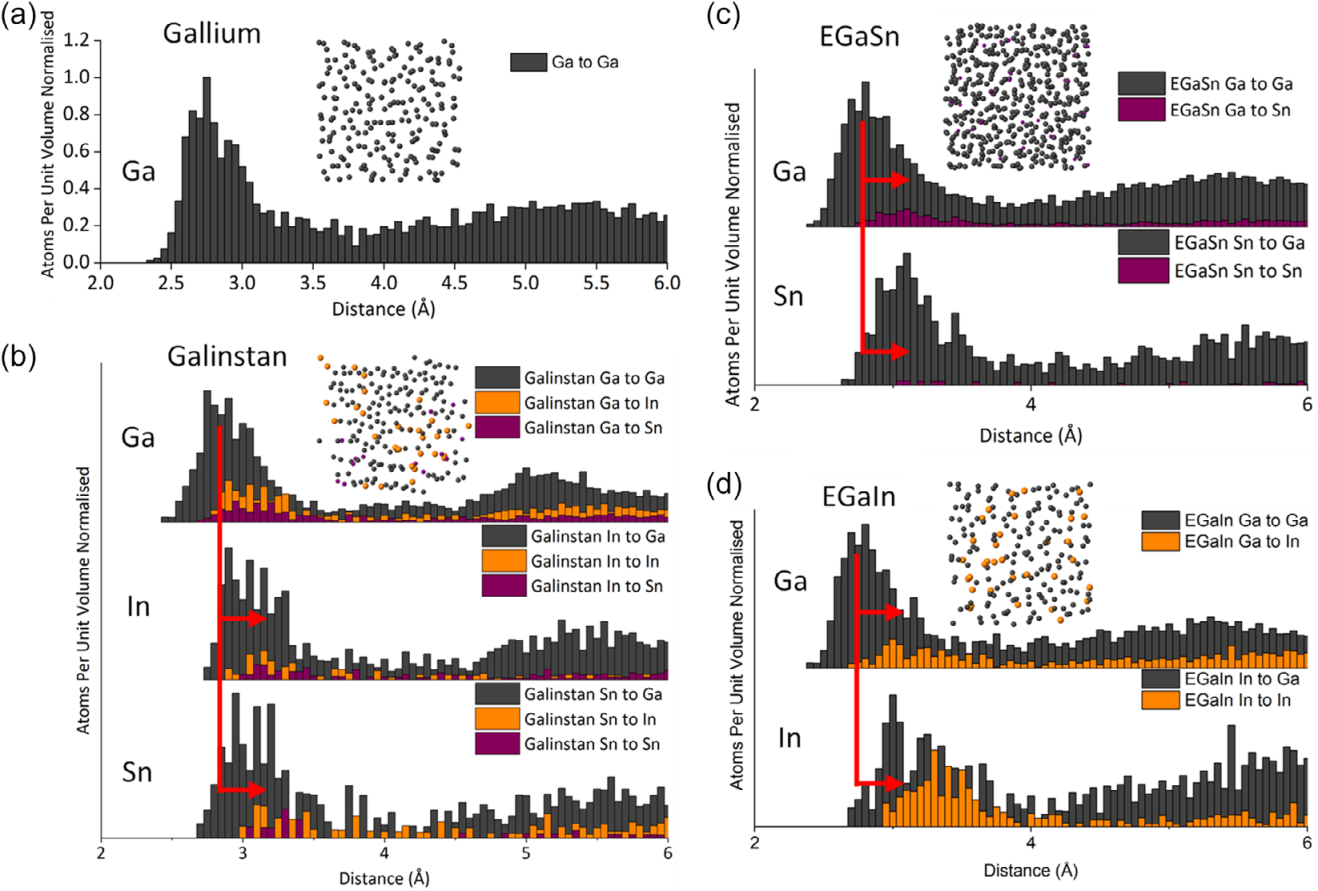
An analysis of the bond length distribution calculated in each of the liquid metal alloys: a) Ga à Ga distances in pure Ga, b) Ga/In/Sn à Ga/In/Sn distances in Galinstan, c) Ga/In à Ga/In distributions in EGaIn, and d) Ga/Sn à Ga/Sn distances in EGaSn.

**Figure 5:**
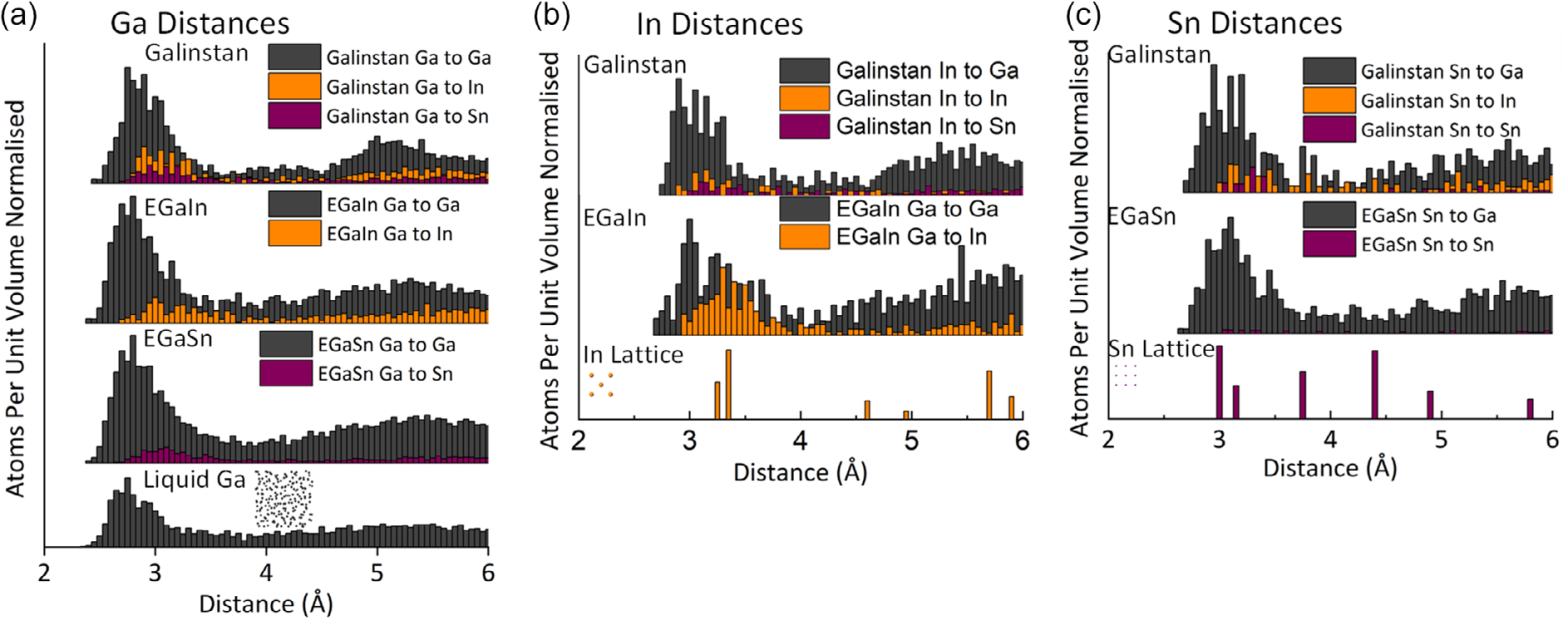
An analysis of the bond length distribution calculated for each element comparing the alloys to each other. The graphs below each section show the distance distribution in the crystal structure of the metals. a) The distribution of distances from Ga atom “out” in Galinstan, EGaIn, EGaSn, and liquid gallium. b) The distribution of distances from the In atoms “out” in EGaIn, Galinstan, and metallic In. c) The distribution of distances from the Sn atoms “out” in Galinstan, EGaSn, and metallic Sn. The data are presented this way to show an average from each element outward, resulting in data comparable to what would be observed in an EXAFS experiment.

While XANES position is indicative of oxidation state, it does have secondary components as well, including effects of coordination number that can alter the location and features of an element's K‐edge. To further investigate this, we also examined the distribution of distances observed from the MD calculations. These are plotted in **Figure** **4** and **5** along with an inset showing the calculated structure. The distances are plotted in two ways which are critical for understanding the alloys and interpreting the XAS. The first plot, Figure 4, compares the distances of each liquid metal and in the second plot Figure 5, we compare each of the elements to each other and the metal crystal structure.

### 2.3 EXAFS Analysis

**Figure 6:**
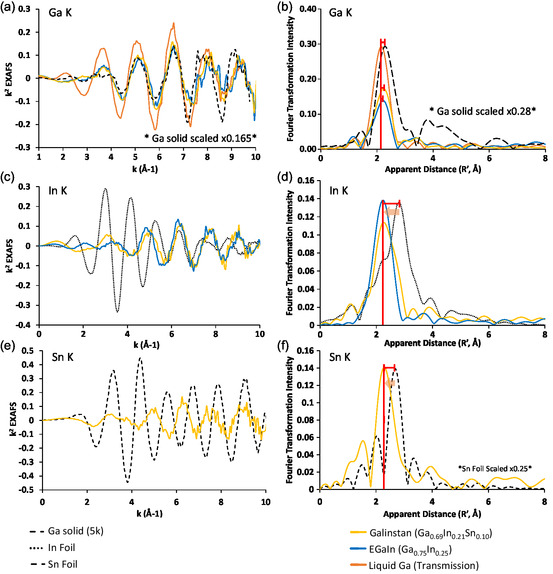
EXAFS (column 1) and FT of the EXAFS (column 2). Color scheme is as follows: gallium as a liquid (orange), gallium frozen in a cryostat (black dashed), In foil (black dotted), Sn foil (black dashed), EGaIn (blue), and Galinstan (yellow) highlighting changes in atomic distances observed at the a,b) Ga, c,d) In, and e,f) Sn K‐edges.

One of the key points to consider with liquid metal alloys is the possible ways that they can mix and bond as outlined in Figure 1. While a homogeneous mixture may occur, other arrangements such as preferential metal–metal bonds, dimers, metal clusters, or even metal colloidal systems may be present in these liquid systems. Three distinct scenarios which can be systematically considered from the EXAFS are illustrated in Figure 1.^[^ ^30^ ^]^ In this study, the EXAFS measurements were performed at each of the three edges in the set of four materials and the data are compared in **Figure** **6**. While there are some exceptions, most metals that share the same bonding structure through the first and second coordination sphere produce EXAFS spectra that are very similar.^[^ ^8^, ^23^ ^]^ This means that if the scenarios illustrated in Figure 1c,d were present, distinct EXAFS spectra would be generated at each of the edges; this, however, is not observed and is consistent with the MD calculations described above.^[^ ^8^ ^]^

Figure 5b and 6a show the EXAFS and Fourier transformation (FT) of the EXAFS at the Ga K‐edge of gallium (black), EGaIn (blue), and Galinstan (yellow). When comparing the EXAFS and FT of the alloys to that of liquid gallium, the FT shows a clear shift to higher apparent distance *R′* in these materials. EGaIn and Galinstan show larger apparent distances at the Ga edge compared to the pure Ga sample. This suggests that in these alloys, atoms surrounding Ga are further away than in metallic Ga. This is likely due to the statistical presence of larger element in the alloy which impacts average distance. Figure 5d and 6c show the EXAFS and FT at the In K‐edge. In Figure 6c, the EXAFS of both Galinstan and EGaIn are similar, indicating that the materials may share a similar first coordination sphere. This is also reflected in the FT (Figure 6d). When comparing the EXAFS and FT to the MD simulations, a similar expansion can be observed. However, while the trends in distance are correct in elements in absolute terms, the distances calculated by MD are substantially longer than those observed by EXAFS. Consistent with what has been noted elsewhere with regard to transition metals,^[^ ^31^ ^]^ this may be important in modeling as 0.06 Å shifts in distances can account for substantial changes in reactivity and density across a material.

FT of the EXAFS at the Sn K‐edge (Figure 6e,f) show a similar smaller first coordination sphere, indicating that when in a liquid state the metal atoms of In and Sn are “on average” closer together than in their solid counterparts.

**Figure 7:**
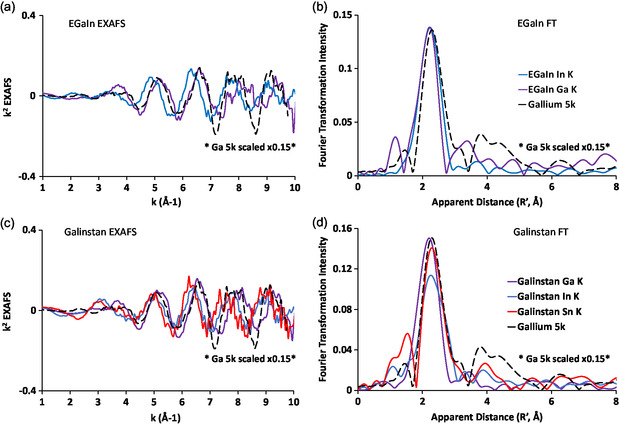
EXAFS (column 1) and FT of the EXAFS (column 2) of the Ga, In, and Sn K‐edges of a,b) EGaIn and c,d) Galinstan showcasing the difference in apparent distances between multiple elements in their respective systems.

The Ga, In, and Sn EXAFS spectra of EGaIn and Galinstan are compared in Figure 6. **Figure** **7**a shows a comparison of the EXAFS and Fourier Transform for the elements in their respective alloys. The figure shows that each EXAFS and FT produces first coordination sphere peaks that are similar in bond length to one another for both EGaIn and in Galinstan.

When examining the EXAFS and the FT of the EXAFS, we considered the possibility of each of the scenarios presented in Figure 1.

When comparing the EXAFS spectra and FT of these systems, it can be concluded that a homogenous mixture of these metals is present due to the similarities shown at the Ga, In, and Sn K‐edges. If other atomic arrangements were present, such as metal dimers, metal‐gallium colloids, or some other biased structure, the FT of these edges would look different at each edge; instead, strikingly similar spectra can be seen at each edge indicating similar bonding environments for all elements. In addition, to fit the EXAFS data well requires a distribution of Ga–Ga contributions not a singular dominant contribution that we might expect for a dimer (see below).

**Figure 8:**
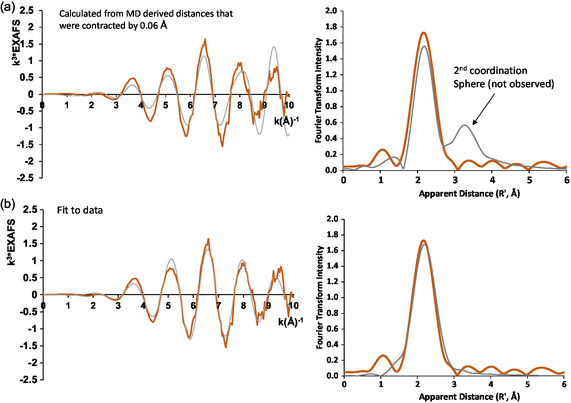
a) EXAFS simulation based on MD calculations where a single distance parameter was floated. The presence of second sphere is observed in MD calculations that is not observed in the experiment presumably because the second coordination sphere is far more dynamic than the first. b) An optimized fit to the EXAFS which included a distribution of Ga distances.

To further study the correlation to the MD simulations, EXAFS fitting was performed and is shown in Figure S3–S6 and Table S1–S3, Supporting Information. One of the challenges of liquid metal systems compared to other systems is that they are not a single structure but rather represent an average of many structures. To account for this and to directly compare the results to the MD simulations, we used a graph bond length frequency throughout the structure to weigh a probability distribution of distances and use that in the fit (Figure S4, Supporting Information). The coordination number or relative contribution of each of the paths was fixed to that determined by the MD calculations so that only the distance parameter and the disorder parameter (the Debye–Waller factor, *σ* ^2^) were floated in the fit. Using only two parameters, a reasonable fit was obtained from the MD calculations; however, the second coordination sphere observed in MD was much stronger than that observed experimentally (**Figure** **8**a). In addition, the bond lengths needed to be contracted by 0.06 Å to match the data, which has implication for how the calculations are working for anything with a distance dependence, including density.

As the second coordination sphere of a structure will have more variation, it is reasonable to assume that the Debye–Waller factors for the fit should have been floated separately. Therefore, we performed a fit where this was the case, and this fit was much improved; however, the Debye–Waller or *σ* ^2^ parameters meant that the second coordination sphere did not contribute substantially to the data. The second coordination sphere observed in MD is likely real; however, the second coordination sphere is likely far more dynamic than the first so the rapid changes in this coordination sphere explain why it is not observed by the XAS.

## 3 Conclusions

This work employed XAS to probe the structural characteristics of liquid Ga, EGaIn, Galinstan, and EGaSn. The results found in this work reveal that these materials exhibit a dynamic and homogeneous distribution of atoms with no signs of short‐range local structure such as dimers under the conditions studied.

The XANES data collected at the In and Sn K‐edge are consistent with an electron transfer trend that follows electronegativity trends of In 1.78, Ga 1.81, and Sn 1.96, respectively. The shifts in the Ga K edge were not consistent with this trend; however, the XANES data were impacted by self‐absorption, making the interpretation of energy shifts challenging. MD simulations were conducted to explore the local structure in these alloys and while the structural trend seen in the MD was consistent with the XAS the simulated atomic distances need to be contracted by 0.06 Å to match the data.

The EXAFS and FT of these samples show several important changes in the atomic structure of these liquid metals. Liquid Ga shows a contraction in its local structure upon melting which is well documented in the literature; however, upon the addition of In and Sn, respectively, a subtle expansion of the local atomic environment from the Ga perspective can be observed. This is opposite to what is observed from the In and Sn perspectives as upon alloying a substantial contraction of the interatomic distances can be observed. When overlaying the atomic distances from each element outward in their respective alloy, the experimental results show each element shares a very similar atomic environment with similar interatomic distances, indicating that these materials exist as homogeneous mixtures and do not possess local structures. This is important because if local structures such as dimers are present it would impact how these metals reacted as catalysts with dimer bonds having to break prior to Ga reacting. As dynamic systems, they are free to react with secondary materials and metals possibly explaining some of the observed catalytic activity.

## 4 Experimental Methods

4.1

4.1.1

##### Preparation of Liquid Metal Alloys

Liquid Ga samples were prepared by heating the wt% ratios of Ga, Sn, and In as follows: EGaIn (Ga 75 wt% and In 25 wt%), EGaSn (Ga 91 wt% and Sn 9 wt%), and Galinstan (Ga 68.5 wt%, In 21.5 wt%, and Sn 10 wt%) and mixing them at 300 °C until a homogeneous mixture is obtained. Following preparation of the alloys, a number of methods were trialed for XAS. To achieve an optimal sample for the technique, several methods were explored including pressing a drop of the liquid metals between X‐Ray transparent tape (Kapton) in an attempt to make a thin layer; loading the liquid metal into a 1 mm diameter quartz capillary by vacuum back filling; and diluting the liquid metals in several agents including BN and cellulose by mechanical grinding in a mortar and pestle with varying concentrations (0.1–10 wt%) and making sodium acetate melts. While commonly used as dilutants in XAS, BN and cellulose did not prove ideal as the material did not disperse as they ordinarily would, as such sodium acetate was used.

##### XAS Analysis

The Australian Synchrotron (AS) is a 3 GeV electron accelerator operating at 200 mA current in top up mode. Measurements for this study were conducted at both the XAS Beamline (ID12) and the Medium Energy X‐Ray XAS (MEX‐1) beamline. The XAS beamline generates X‐rays from a 1.9 T multipole wiggler and uses a liquid nitrogen cooled double crystal monochromator. A set of Si (111) crystals were used to collect data at the Ga K‐edge while data for the In and Sn K‐edges were collected using a pair of Si (311) crystals. MEX‐1 beamline generates X‐rays from a bending magnet generating a magnetic field of 1.3T and uses a liquid cooled double crystal monochromator. Similar to XAS, a pair of Si (111) crystals were used to collect data at the Ga K‐edge. Samples were placed in a He gas environment, with data collected in both fluorescence and transmission modes simultaneously at MEX‐1 and XAS. Data collected at XAS were done using OKEN ionization chambers filled with N_2_ gas. The samples were placed at a 45° to the beam. Data were collected in triplicate at each edge and averaged and processed using a combination of Athena,^[27a]^ Artemis,^[27a]^ and Excel.^[^ ^32^ ^]^ The Ga K‐edge data were calibrated^[^ ^33^ ^]^ to the inflection point of the first derivative for metallic gallium at 10,367.1 eV. Likewise, indium (In) and tin (Sn) were calibrated to the first inflexion points of their foils of 27,939.9 and 29,200.1 eV, respectively. *k* = 0 or E0 for the EXAFS spectra presented was defined as 27 954 eV for In, 29 217 eV for Sn, and 10 386 eV for Ga.

##### Self‐Absorption Corrections

Self‐absorption corrections were conducted using Athena, which implements the method described by Troger et al.^[27c]^ and Goulon et al.;^[27b]^ material compositions were entered using each sample's wt% makeup. Figure S2, Supporting Information shows a self‐absorption simulation of various dilutions of Ga, In, and Sn; this was done by altering the sample composition in Athena to include the dilutant and varying the % of metal to dilutant. It is notable that the simulations do a good job of correcting the self‐absorption effects observed at the In and Sn edge but less well at the Ga edge. Simulations were also experimental observations as well as by direct material formulation as effects calculated with the correct material formation were of a lesser magnitude than those observed experimentally.

##### MD Simulations

Initial structures for the ab initio MD simulations of the four bulk Ga‐based systems were adapted from the previous work,^[^ ^34^ ^]^ with an equilibrated snapshot of 500 Ga atoms in a cubic box used as a starting point for all simulations. The initial dimensions for the Ga system were (21.198 × 21.198 × 21.198) Å^3^ to reproduce the bulk density of liquid Ga at 30 °C.

The box lengths were changed to 21.213 Å to reflect the change in density of the Ga system at 50 °C. The EGaIn system was created by randomly changing 82 bulk Ga atoms to In and increasing the box lengths to 21.740 Å. For Galinstan, 7 In and 27 Ga were randomly changed to Sn and the box lengths modified to 21.766 Å. For the EGaSn system, 27 Ga atoms were randomly changed to Sn atoms and the box lengths to 21.414 Å. Multiple initial random configurations of In and Sn were investigated.

All simulations were carried out using the Vienna Ab initio Simulation Package^[^ ^35^ ^]^ at 323.15 K with the projector‐augmented wave method,^[^ ^36^ ^]^ the PBE exchange–correlation functional,^[^ ^37^ ^]^ and an energy cutoff of 150 eV. For all systems, only the gamma point was used to sample the Brillouin zone. Bulk systems were run for 50 ps, with an initial 5 ps of equilibration using a velocity rescale thermostat, followed by production runs with the Nose–Hoover thermostat. All analysis was performed using VMD 1.9.3.^[^ ^38^ ^]^

The Bader charge calculations were initialized by performing a geometry optimization on the final snapshot of the MD simulations. The final, optimized frames were then used to run the Bader charge calculations within the system.

## Conflict of Interest

The authors declare no conflict of interest.

## Author Contributions

**Torben Daeneke**: Methodology (equal); Supervision (equal); Writing—original draft (supporting); Writing—review and editing (supporting). **Jaydon A. Meilak**: Conceptualization (equal); Data curation (lead); Formal analysis (equal); Investigation (equal); Methodology (lead); Writing—original draft (lead); Writing—review and editing (lead). **Karma Zuraiqi**: Conceptualization (equal); Methodology (equal); Resources (equal); Validation (supporting); Writing—review and editing (supporting). **Valerie Mitchell**: Conceptualization (supporting); Methodology (supporting); Resources (equal); Software (equal); Validation (supporting); Writing—review and editing (supporting). **Bernt Johannessen**: Conceptualization (supporting); Data curation (supporting); Methodology (supporting); Resources (equal); Supervision (supporting). **Brittany V. Kerr**: Formal analysis (supporting); Investigation (supporting); Validation (supporting); Writing—review and editing (supporting). **Pierre H. A. Vaillant**: Data curation (equal); Formal analysis (equal); Investigation (supporting); Methodology (supporting); Writing—review and editing (supporting). **Krystina Lamb**: Conceptualization (supporting); Data curation (supporting); Resources (supporting); Software (supporting); Supervision (supporting); Writing—review and editing (supporting). **Patjaree Aukarasereenont**: Data curation (supporting); Methodology (equal); Resources (equal); Writing—review and editing (supporting). **Caiden Parker**: Data curation (supporting); Methodology (equal); Resources (equal); Writing—review and editing (supporting). **Taren Cataldo**: Validation (equal); Writing—review and editing (equal). **Francois Malherbe**: Supervision (equal); Validation (equal); Writing—review and editing (equal). **Andrew J. Christofferson**: Data curation (equal); Formal analysis (equal); Investigation (equal); Methodology (equal). **Rosalie K. Hocking**: Conceptualization (equal); Formal analysis (equal); Investigation (equal); Methodology (equal); Project administration (equal); Supervision (equal); Validation (equal); Writing—original draft (equal); Writing—review and editing (equal).

## Supporting information

Supplementary Material

## Data Availability

The data that support the findings of this study are available from the corresponding author upon reasonable request.
