# Correction to “An X‐Ray Absorption Spectroscopy Investigation into the Fundamental Structure of Liquid Metal Alloys”

**DOI:** 10.1002/smsc.202400581

**Published:** 2024-12-06

**Authors:** Jaydon A. Meilak, Karma Zuraiqi, Valerie Mitchell, Bernt Johannessen, Brittany V. Kerr, Pierre H. A. Vaillant, Krystina Lamb, Patjaree Aukarasereenont, Caiden Parker, Taren Cataldo, Francois Malherbe, Andrew J. Christofferson, Torben Daeneke, Rosalie K. Hocking

*Small Sci*. **2024**, *4*, 2400317, https://doi.org/10.1002/smsc.202400317

It is noted that the affiliation recorded for Valerie Mitchell, Bernt Johannessen, and Krystina Lamb is incomplete. The full affiliation should be ‘Australian Synchrotron, ANSTO, Clayton VIC 3168, Australia’. We apologise for any inconvenience caused. The correction does not in any way affect results or conclusions of the article.

