# Supplementary material for: An X‐Ray Absorption Spectroscopy Investigation into the Fundamental Structure of Liquid Metal Alloys
Source: Small Sci. 2024 Sep 23;4(11):2400317. doi: 10.1002/smsc.202400317 (PMC11935233; doi:10.1002/smsc.202400317)
Supplement: Supplementary file 1 — Supplementary Material [file SMSC-4-2400317-s001.pdf]

Supporting Information for:

An XAS Investigation into the fundamental structure of Liquid Metal Alloys

Jaydon A Meilak[1], Karma Zuraiqi[2], Valerie Mitchell[3], Bernt Johannessen[3], Brittany V Kerr[1], Pierre H A Vaillant[2], Krystina Lamb[3], Patjaree Aukarasereenont[2], Caiden Parker[2], Taren Cataldo[1], Francois Malherbe[1], Andrew J. Christofferson[2], Torben Daeneke[2,*], Rosalie K. Hocking[1,*]

[1]Swinburne University of Technology, John St Hawthorn Australia VIC 3122

[2]Royal Melbourne Institute of Technology, 124 Latrobe St Melbourne Australia VIC 3000

[3]Australian Synchrotron, 800 Blackburn Rd Clayton Australia VIC 3168

1. Discussion of Sample and Spectral consideration in studying liquid metals

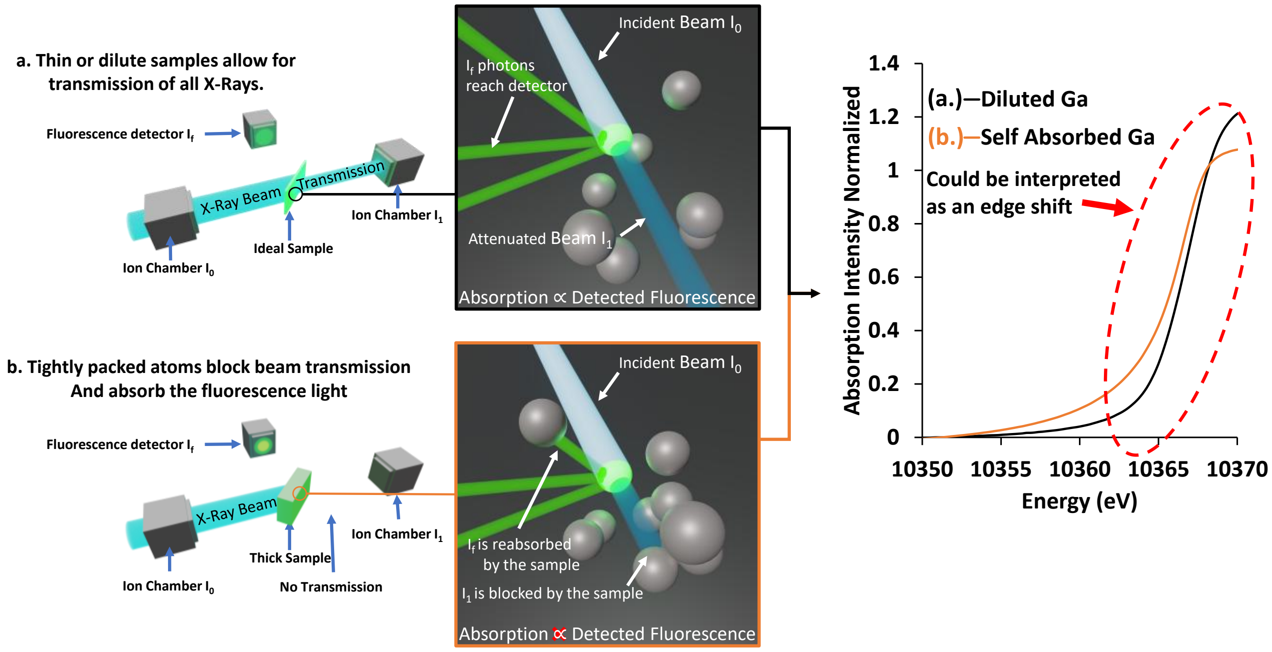

Figure S1. effects of sample concentration or thickness on XANES spectra highlighting how highly concentrated or thick samples (b.) can alter the signal proportionality of light in/out vs a thin or diluted sample (a.) resulting in data that can appear falsely shifted.

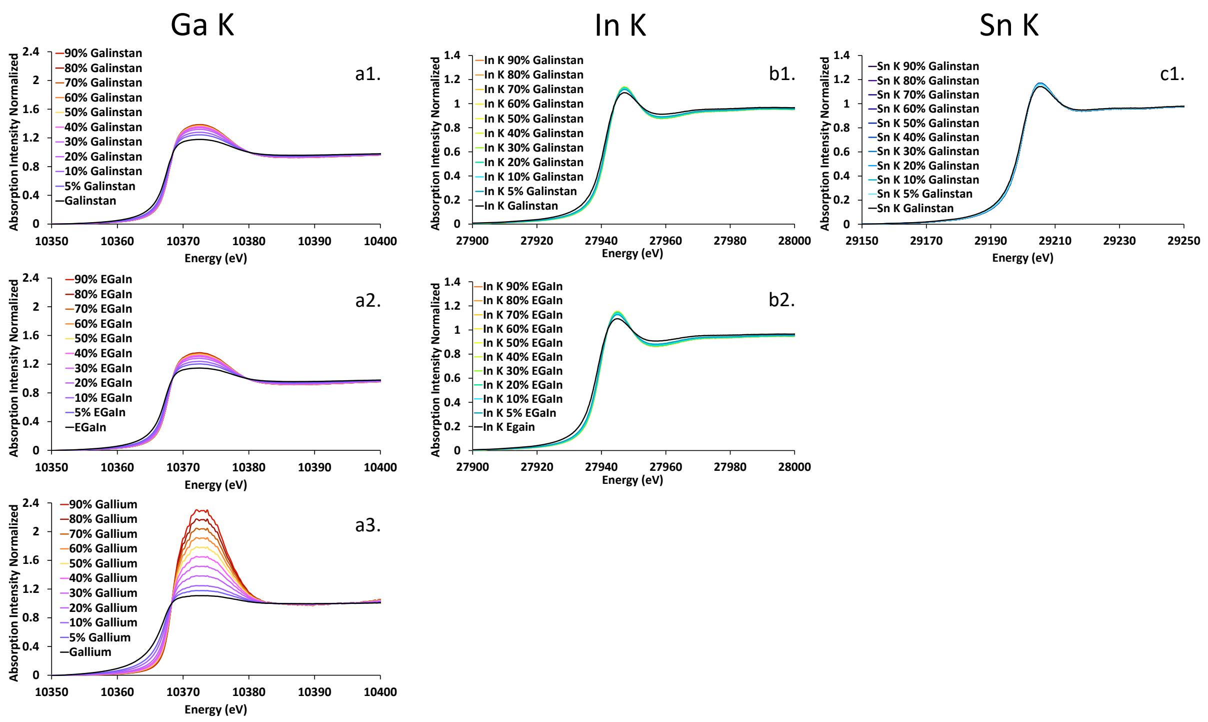

Figure S2. Simulated self-absorption correction of the Ga, In, and Sn k edges of pure Ga, EGaIn, and Galinstan. SA simulations were done by altering the composition of pure metal and metal alloy scans to include a dilutant and then varying the ratios of dilutant to sample.

While we could obtain data on the liquid metal samples it is important that we discuss some of the challenges we encountered in doing this. There are two ways X-ray absorption data can be collected in fluorescence mode and in transmission mode. (See Figure S1) In order to collect transmission data samples need to sufficiently spectrally diluted with a low Z material to allow for both the incident beam and fluorescence signal to pass through the sample without being hindered (Figure S1a). The hinderance of the outgoing light is referred to as self-absorption and can lead to false readings such as oxidation stat or Z_{eff} shifts (Figure S1b). During these experiments, we did attempt to dilute our samples with common XAS dilutants such as BN and cellulose. However, both were found to react with liquid metals. We also attempted to manufacture thin films by pressing the sample between two pieces of Kapton tape however the surface tension of Ga did not allow for the sample thickness to be uniform throughout the sample. As such much of the data shown here is fluorescence data. In fluorescence experiment we fundamentally assume that the fluorescent X-rays are proportional to the absorbed X-rays. However, this assumption fails in the thick samples because some of the fluorescent X-rays are reabsorbed by the sample (Figure S1b). To some

extent we could not avoid collecting self-absorbed data one of the assumptions we make above is that all samples are to first approximation equally self-absorbed. While self-absorption can be corrected for mathematically (Si Figure 2) we chose not to use this in our work as the process seemed to overcorrect for this when dealing with high concentration samples like the Ga K edge, we instead looked at these scans and used the trend seen from the simulated data to help inform us on the overall trend of the Ga K edge.

2. EXAFS Fit to crystallographically defined Ga.

In order to understand the structure Ga, the EXAFS collected at at temperature of 10 K was compared to that published crystal structure of metallic Gallium described by Sharma and Donahue[1]

In order to assess this fit, a limited parameter set which floated the shortest distances as one group (indicated in the grey shading) in Table S1 and the remaining distances are a second group.

Table S1. Fit Parameter for Frozen Gallium from the Crystal structure reported by Sharma et al.,

Distance (Ga-Ga)	R(fit)	R(error)	N coord no.	s^2	±
**Group 1**					
2.48	2.47	0.011	1	0.0055	0.0017
2.69	2.67	0.011	2	0.0055	0.0017
2.73	2.71	0.011	2	0.0055	0.0017
2.79	2.77	0.011	2	0.0055	0.0017
**Group 2**					
3.75	3.71	0.032	2	0.0164	0.0041
3.98	3.94	0.032	4	0.0164	0.0041
4.1	4.06	0.032	6	0.0164	0.0041
4.1	4.06	0.032	6	0.0164	0.0041
4.12	4.08	0.032	2	0.0164	0.0041
4.45	4.41	0.032	5	0.0164	0.0041
4.52	4.48	0.032	4	0.0164	0.0041
4.52	4.48	0.032	4	0.0164	0.0041
4.65	4.6	0.032	2	0.0164	0.0041

Other parameters E0 -15.9± 1.7; S02 =0.779 ± 0.164 R= 0.076

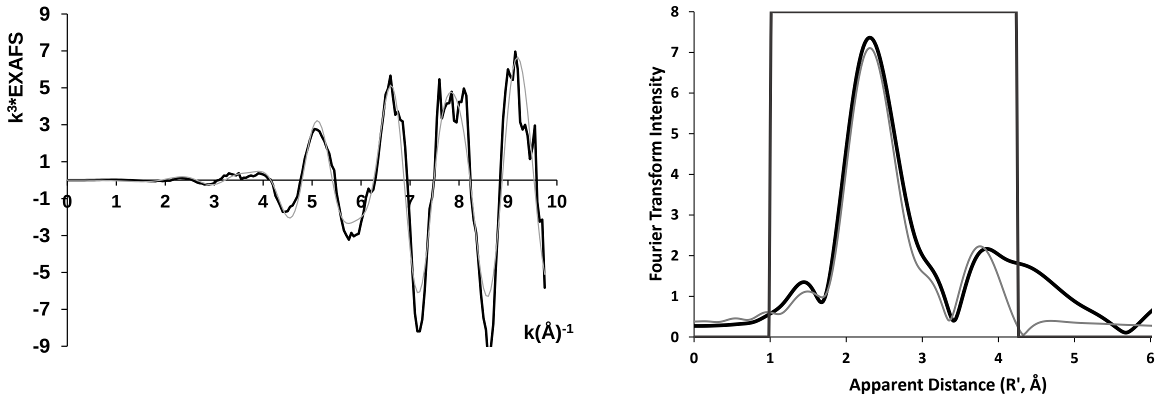

Figure S3. Fit Crystalline Ga with parameters shown in Table S1.

3. Comparison of MD simulations to the EXAFS data

To compare the EXAFS fits to the simulations several strategies were examined. One of the challenges in a liquid we see the sum of all possible sites originating from Ga simultaneously so if we calculate the EXAFS from a single site, it might not be representative of the average of all sites. To accommodate this we took output from the MD calculation and made a histogram of all bonds in 0.05 Å increments shown in Figure S4.

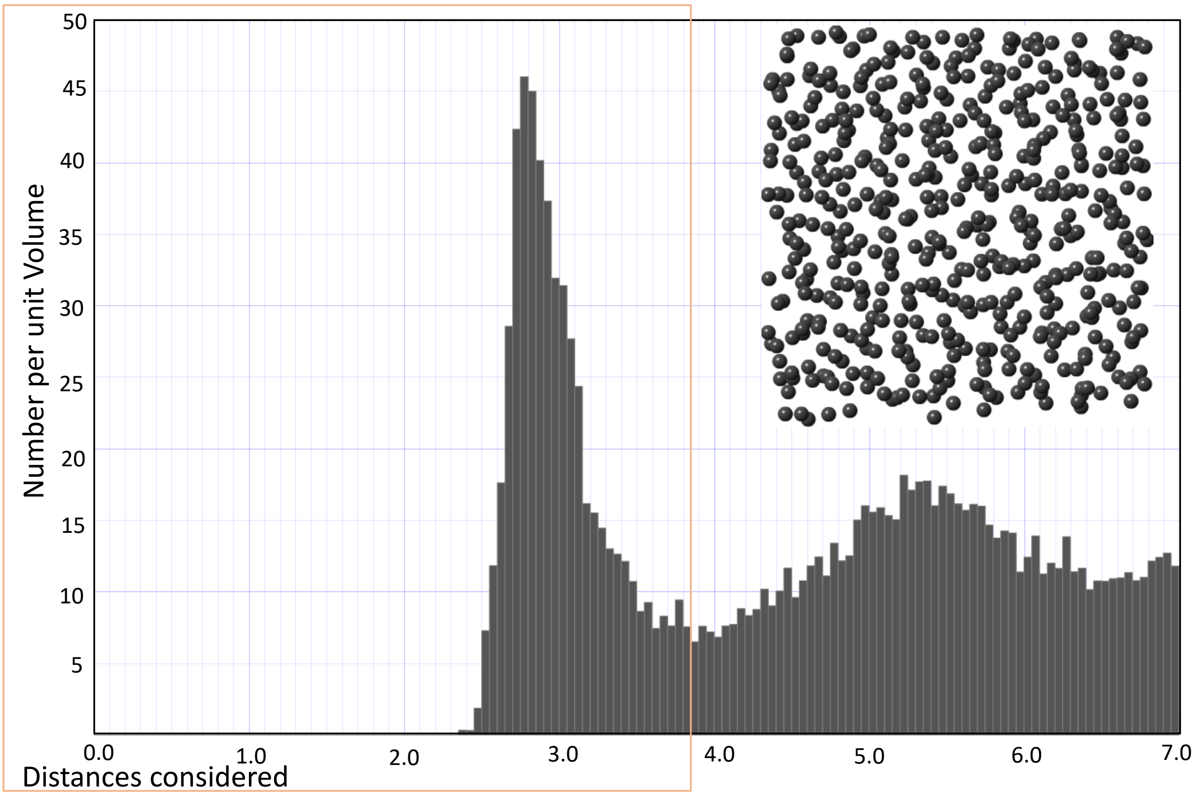

Figure S4. The MD simulation of Ga and the histogram of distances used to simulate the EXAFS.

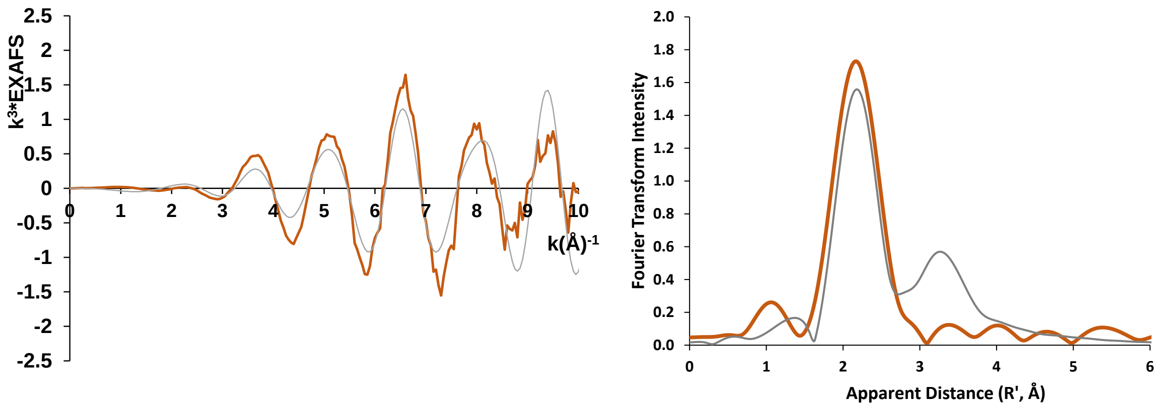

Figure S5. EXAFS fit with the distribution of distances calculated in the MD. The Debye Waller, s2 parameter was the same for all values.

Table S2. Parameters used for the EXAFS fit shown in Figure S5.

int.	Distance (Å)	N (Calculated)**	SO2 (set)	s^2 (floated as 1 set)	Ds^2	E0	DE0	DR	DDR	R, fit
**Ga-Ga**	2.5	0.16	0.6	0.0036	0.0014	-12.2	3	0.059	0.02	2.56
**Ga-Ga**	2.6	0.25	0.6	0.0036	0.0014	-12.2	3	0.059	0.02	2.61
**Ga-Ga**	2.6	0.54	0.6	0.0036	0.0014	-12.2	3	0.059	0.02	2.66
**Ga-Ga**	2.7	0.86	0.6	0.0036	0.0014	-12.2	3	0.059	0.02	2.71
**Ga-Ga**	2.7	0.75	0.6	0.0036	0.0014	-12.2	3	0.059	0.02	2.76
**Ga-Ga**	2.8	0.73	0.6	0.0036	0.0014	-12.2	3	0.059	0.02	2.81
**Ga-Ga**	2.8	0.79	0.6	0.0036	0.0014	-12.2	3	0.059	0.02	2.86
**Ga-Ga**	2.9	0.65	0.6	0.0036	0.0014	-12.2	3	0.059	0.02	2.91
**Ga-Ga**	2.9	0.78	0.6	0.0036	0.0014	-12.2	3	0.059	0.02	2.96
**Ga-Ga**	3	0.66	0.6	0.0036	0.0014	-12.2	3	0.059	0.02	3.01
**Ga-Ga**	3	0.56	0.6	0.0036	0.0014	-12.2	3	0.059	0.02	3.06
**Ga-Ga**	3.1	0.6	0.6	0.0036	0.0014	-12.2	3	0.059	0.02	3.11
**Ga-Ga**	3.1	0.41	0.6	0.0036	0.0014	-12.2	3	0.059	0.02	3.16
**Ga-Ga**	3.2	0.38	0.6	0.0036	0.0014	-12.2	3	0.059	0.02	3.21
**Ga-Ga**	3.2	0.44	0.6	0.0036	0.0014	-12.2	3	0.059	0.02	3.26
**Ga-Ga**	3.3	0.38	0.6	0.0036	0.0014	-12.2	3	0.059	0.02	3.31
**Ga-Ga**	3.3	0.33	0.6	0.0036	0.0014	-12.2	3	0.059	0.02	3.36
**Ga-Ga**	3.4	0.37	0.6	0.0036	0.0014	-12.2	3	0.059	0.02	3.41
**Ga-Ga**	3.4	0.18	0.6	0.0036	0.0014	-12.2	3	0.059	0.02	3.46
**Ga-Ga**	3.5	0.24	0.6	0.0036	0.0014	-12.2	3	0.059	0.02	3.51
**Ga-Ga**	3.5	0.12	0.6	0.0036	0.0014	-12.2	3	0.059	0.02	3.56
**Ga-Ga**	3.6	0.27	0.6	0.0036	0.0014	-12.2	3	0.059	0.02	3.61
**Ga-Ga**	3.6	0.27	0.6	0.0036	0.0014	-12.2	3	0.059	0.02	3.66
**Ga-Ga**	3.7	0.38	0.6	0.0036	0.0014	-12.2	3	0.059	0.02	3.71

**This N is coordination number, this was based on the calculated distribution of distances from the MD simulations and was set for the EXAFS calculations

***SO2 is related to N as these parameters are complicated by the dynamic nature of these systems they were set as the same value.

**** Even though this table looks like there are a lot of parameters to facilitate comparison to the MD only the DR and a single Debye Waller parameters were floated

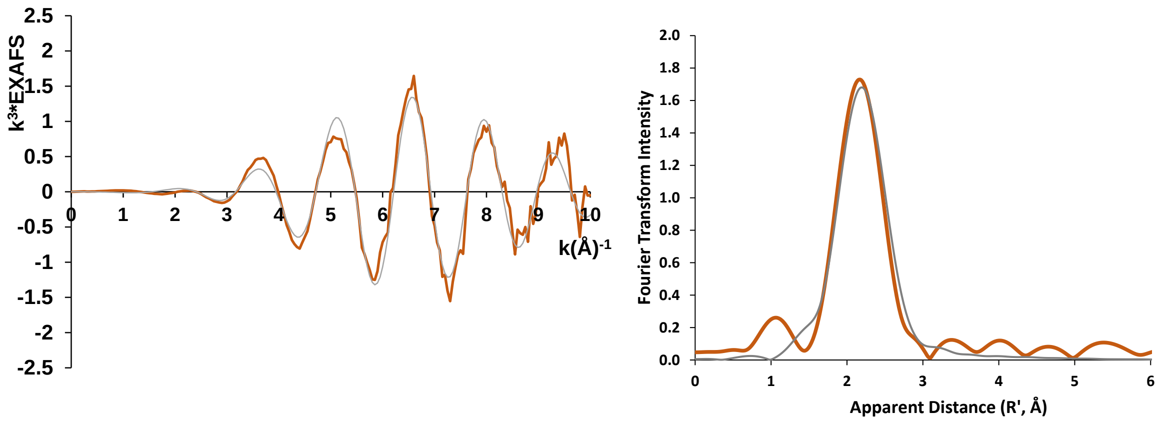

Figure S6. EXAFS fit with the distribution of distances calculated in the MD. The Debye Waller, s2 parameter was the same for all distances between 2.5 and 2.8 a second s2 parameter was introduced for distances between 2.8 and 3.7.

Table S3. Parameters used for the EXAFS fit shown in Figure SZ.

**Int.**	**Distance (Å)**	**N (Calculated)****	**SO2 (set)**	**s^2 (floated as 2 sets)**	**Ds2**	**E0**	**DE0**	**DR**	**DDR**	**R, fit**
**Ga-Ga**	2.5	0.16	0.6	0.0076	0.0005	-20.54	1.17	-0.009	0.01	2.49
**Ga-Ga**	2.6	0.25	0.6	0.0076	0.0005	-20.54	1.17	-0.009	0.01	2.54
**Ga-Ga**	2.6	0.54	0.6	0.0076	0.0005	-20.54	1.17	-0.009	0.01	2.59
**Ga-Ga**	2.7	0.86	0.6	0.0076	0.0005	-20.54	1.17	-0.009	0.01	2.64
**Ga-Ga**	2.7	0.75	0.6	0.0076	0.0005	-20.54	1.17	-0.009	0.01	2.69
**Ga-Ga**	2.8	0.73	0.6	0.0076	0.0005	-20.54	1.17	-0.009	0.01	2.74
**Ga-Ga**	2.8	0.79	0.6	0.0076	0.0005	-20.54	1.17	-0.009	0.01	2.79
**Ga-Ga**	2.9	0.65	0.6	0.3264	0.9	-20.54	1.17	-0.009	0.01	2.84
**Ga-Ga**	2.9	0.78	0.6	0.3264	0.9	-20.54	1.17	-0.009	0.01	2.89
**Ga-Ga**	3	0.66	0.6	0.3264	0.9	-20.54	1.17	-0.009	0.01	2.94
**Ga-Ga**	3	0.56	0.6	0.3264	0.9	-20.54	1.17	-0.009	0.01	2.99
**Ga-Ga**	3.1	0.6	0.6	0.3264	0.9	-20.5	1.17	-0.00	0.01	3.04

						4		9		
**Ga-Ga**	3.1	0.41	0.6	0.3264	0.9	-20.54	1.17	-0.009	0.01	3.09
**Ga-Ga**	3.2	0.38	0.6	0.3264	0.9	-20.54	1.17	-0.009	0.01	3.14
**Ga-Ga**	3.2	0.44	0.6	0.3264	0.9	-20.54	1.17	-0.009	0.01	3.19
**Ga-Ga**	3.3	0.38	0.6	0.3264	0.9	-20.54	1.17	-0.009	0.01	3.24
**Ga-Ga**	3.3	0.33	0.6	0.3264	0.9	-20.54	1.17	-0.009	0.01	3.29
**Ga-Ga**	3.4	0.37	0.6	0.3264	0.9	-20.54	1.17	-0.009	0.01	3.34
**Ga-Ga**	3.4	0.18	0.6	0.3264	0.9	-20.54	1.17	-0.009	0.01	3.39
**Ga-Ga**	3.5	0.24	0.6	0.3264	0.9	-20.54	1.17	-0.009	0.01	3.44
**Ga-Ga**	3.5	0.12	0.6	0.3264	0.9	-20.54	1.17	-0.009	0.01	3.49
**Ga-Ga**	3.6	0.27	0.6	0.3264	0.9	-20.54	1.17	-0.009	0.01	3.54
**Ga-Ga**	3.6	0.27	0.6	0.3264	0.9	-20.54	1.17	-0.009	0.01	3.59
**Ga-Ga**	3.7	0.38	0.6	0.3264	0.9	-20.54	1.17	-0.009	0.01	3.64

MD simulation results:

Table S4. MD simulations of Galinstan

**Galinstan**				
	**1**	**2**	**3**	**Tot**
**All**	0.0000	0.0000	0.0000	0.0000
**Ga**	-0.0297	-0.0314	-0.0314	-0.0308
**In**	0.1414	0.1461	0.1440	0.1438
**Sn**	0.0316	0.0404	0.0462	0.0394
**In and Sn**	0.1079	0.1138	0.1141	0.1119
**Ga < 3.3 from In**	-0.0360	-0.0368	-0.0369	-0.0366
**In < 3.3 from Ga**	0.1093	0.1136	0.1141	0.1123
**Together**	0.0024	0.0021	0.0023	0.0023
**Ga < 3.3 from Sn**	-0.0394	-0.0416	-0.0400	-0.0403
**Sn < 3.3 from Ga**	0.1414	0.1462	0.1440	0.1439
**Together**	0.0031	0.0026	0.0024	0.0027
**Ga < 3.3 from In & Sn**	-0.0347	-0.0355	-0.0376	-0.0360
**In & Sn < 3.3 from Ga**	0.0338	0.0404	0.0462	0.0401
**Together**	-0.0210	-0.0220	-0.0226	-0.0219
**In < 3.3 from Sn**	0.1261	0.1396	0.1364	0.1340
**Sn < 3.3 from In**	0.0046	0.0241	0.0172	0.0153
**Together**	0.0721	0.0857	0.0791	0.0790
**Ga > 3.3 from In**	-0.0099	-0.0119	-0.0115	-0.0111
**Ga > 3.3 from Sn**	-0.0137	-0.0152	-0.0163	-0.0151
**Ga > 3.3 from In & Sn**	-0.0273	-0.0287	-0.0275	-0.0279
**In > 3.3 from Sn**	0.1411	0.1501	0.1404	0.1439
**Sn > 3.3 from In**	0.0570	0.0525	0.0627	0.0574

Table S5. MD simulations of Gallium

**Gallium**				
	**1**	**2**	**3**	**Tot**
**Ga/All**	0.0000	0.0000	0.0000	0.0000

Table S6. MD simulations of EGaIn

**EGaIn**				
	**1**	**2**	**3**	**Tot**
**All**	0.0000	0.0000	0.0000	0.0000
**Ga**	-0.0299	-0.0272	-0.0301	-0.0291
**In**	0.1526	0.1384	0.1537	0.1482
**Ga < 3.3 from In**	-0.0407	-0.0374	-0.0381	-0.0387
**In < 3.3 from Ga**	0.1526	0.1385	0.1537	0.1483
**Together**	0.0054	0.0051	0.0049	0.0051
**Ga > 3.3 from In**	-0.0119	-0.0113	-0.0134	-0.0122

Table S7. MD simulations of EGaSn

**EGaSn**				
	**1**	**2**	**3**	**Tot**
**All**	0.0000	0.0000	0.0000	0.0000
**Ga**	-0.0043	-0.0045	-0.0046	-0.0045
**Sn**	0.0760	0.0796	0.0804	0.0787
**Ga < 3.3 from Sn**	-0.0100	-0.0109	-0.0130	-0.0113
**Sn < 3.3 from Ga**	0.0760	0.0796	0.0804	0.0787
**Together**	0.0030	0.0029	0.0011	0.0023
**Ga > 3.3 from Sn**	-0.0017	-0.0016	-0.0006	-0.0013

References:

[1] B. D. Sharma and J. Donohue, *Zeitschrift für Kristallographie* **1962**, *117*, 293-300.

Contents

Discussion of Sample and Spectral consideration in studying liquid metals 2

Figure S1. effects of sample concentration or thickness on XANES spectra 2

Figure S2. Simulated self-absorption correction of the Ga k edge 3

EXAFS Fit to crystallographically defined Ga 4

Table S1. Fit Parameter for Frozen Gallium from the Crystal structure reported by Sharma et al., ... 4

Figure S3. Fit Crystalline Ga with parameters shown in Table S1. 5

Comparison of MD simulations to the EXAFS data 5

Figure S5. EXAFS fit with the distribution of distances calculated in the MD. The Debye Waller, s2 parameter was the same for all values. 6

Si Table 2. Parameters used for the EXAFS fit shown in Figure S5. 7

Figure S6. EXAFS fit with the distribution of distances calculated in the MD. 8

Table S3. Parameters used for the EXAFS fit shown in Figure SZ. 9

MD simulation results: 10

Table S4. MD simulations of Galinstan 10

Table S5. MD simulations of Gallium 11

Table S6. MD simulations of EGaIn 11

Table S7. MD simulations of EGaSn 11
